# Spine gourd (*Momordica dioica* Roxb.): an orphan climbing vine attaining new heights due to its healthcare properties

**DOI:** 10.3389/fphar.2026.1761413

**Published:** 2026-03-25

**Authors:** Suchismita Chatterjee, Preeti Sagar, Ashish Kumar Tiwari, Devendra Upadhyay, Sonal Tiwari, Anjali Singh, Bhavna Tiwari, Anjali Sharma, Yogendra Kumar Mishra, Rumana Khan, Ashutosh Singh, Jitendra Kumar Tiwari, Anil Kumar

**Affiliations:** 1 Indira Gandhi Krishi Vishwavidyalaya, Raipur, Chhattisgarh, India; 2 Mahatma Gandhi Horticultural and Forestry University, Durg, Chhattisgarh, India; 3 Rani Lakshmi Bai Central Agricultural University, Jhansi, Uttar Pradesh, India

**Keywords:** functional food, medicinal plant, *Momordica dioica*, nutritional benefits, spine gourd

## Abstract

Spine gourd (*Momordica dioica* Roxb.) is a dioecious perennial climber traditionally consumed in South and Southeast Asia and valued in indigenous dietary practices and traditional medicine. Despite its high nutritional value, adaptability, and well-documented ethnomedicinal relevance, the crop remains underutilized in mainstream agriculture and functional food systems, resulting in a significant knowledge and utilization gap. At a time when plant-based foods are increasingly explored to address metabolic disorders, micronutrient deficiencies, and chronic inflammatory conditions, *Momordica dioica* offers considerable promise as a nutrient-dense vegetable. The fruit contains significant amount of protein (≈18–19%), dietary fiber (≈21–22%), carbohydrates (≈45–48%), and minerals such as calcium (33–35 mg/100 g), iron (4–5 mg/100 g), and phosphorus (42–45 mg/100 g), among other essential nutrients. Emerging scientific studies support several traditional claims of historical uses of the plant parts, reporting antioxidant, antidiabetic, anti-inflammatory, antimicrobial, nephroprotective, and anticancer activities associated with phytochemicals such as triterpenoids, flavonoids, sterols, saponins, and phenolic compounds. However, existing knowledge is dispersed across agronomic, phytochemical, and pharmacological studies, limiting a cohesive understanding of its full potential as both a food crop and therapeutic resource. This review consolidates the current information on taxonomy, distribution, botanical features, cultivation practices, phytochemistry, traditional uses, and experimentally validated bioactivities, thereby addressing fragmentation in the literature. In conclusion, the study positions spine gourd as an underexploited yet valuable crop with strong potential for mainstream vegetable production, improved nutritional security, sustainable agriculture, and future plant-based health applications.

## Introduction

Spine gourd (*Momordica dioica* Roxb.), an underutilized but nutritionally and economically relevant cucurbit, is gradually emerging as a crop of interest in Indian and Southeast Asian agriculture. While it is not as widely recognized as other species within the *Momordica* genus—which includes approximately 60 taxa, predominantly distributed across tropical regions of the Old World (Schaefer and Renner, 2010; Asna et al., 2020). This species has demonstrated strong adaptability to diverse tropical and subtropical environments and possesses a rich profile of phytochemicals. Most species in this genus grow as annual or perennial vines and display distinctive floral features, such as the secretion of floral oils that attract specialized pollinators from the Ctenoplectrini tribe of Hymenoptera (sub family Apinae) (Schaefer and Renner, 2010; Asna et al., 2020).

In recent years, spine gourd has gained significant research interest for its potential role in enhancing nutritional security and supporting farm-based incomes, especially in regions with limited agricultural resources and rain dependent systems. Several genotypes have shown promising yield performance, producing between 600 and 650 g per plant under favourable growing conditions (Singh et al., 2008). The crop is increasingly being grown in plains, peri-urban zones, and various tropical agro climatic regions. It is recognized for its high content of protein, calcium, phosphorus, iron, and carotene being the highest recorded among cucurbits, which contributes significantly to building immunity and nutritional adequacy. The expansion in cultivation is being driven by increasing market demand, owing to its recognized medicinal uses, long shelf life, and its suitability for long-distance transportation and export (Rasul, 2003). At present, West Bengal and Karnataka are among the leading states in India to be engaged in commercial cultivation of the crop. Improved varieties such as Indira Kankoda I (RMF 37) have shown adaptability across a wide range of Indian states, including Uttar Pradesh, Odisha, Maharashtra, Jharkhand, Chhattisgarh, and parts of Meghalaya (Kumar et al., 2024). Reported yields for varieties like Indira Kankoda 1, Indira Kankoda 2, and Chhattisgarh Kankoda 2 are in the range of 30–45 quintals per hectare (Tiwari et al., 2022b). A characterization study of genetic resources by Chatterjee et al., 2023, has demonstrated considerable yield variability among spine gourd genotypes. The study reported an average fruit yield of 1.26 kg per plant with several high yielding lines. Among the checks, Indira Kankoda 1 recorded about 100 fruits per plant with an average fruit weight of 20.44 g (≈2.04 kg per plant), Indira Kankoda 2 produced 113 fruits with a mean weight of 19.78 g (≈2.23 kg per plant), while Chhattisgarh Kankoda 2 yielded about 91 fruits averaging 18.85 g (≈1.71 kg per plant). In addition, several mutant lines outperformed these checks for yield and yield contributing traits, highlighting the availability of high-performing genotypes suitable for genetic improvement and commercial exploitation.

Despite these advantages, large-scale cultivation of spine gourd remains constrained due to its vegetative propagation mode and dioecious nature, which require careful management of male-to-female plant ratios. The crop is propagated primarily through tubers or stem cuttings, which restrict scalability due to limited availability of propagules and low multiplication rates. While cultivation through seeds is possible, it is often avoided because of erratic germination, dormancy, and unpredictable sex expression (Mondal et al., 2006). Moreover, male plants tend to dominate wild populations, and sex differentiation is only possible after flowering, further complicating seed-based propagation. Commercial fields thus need to include around 5%–10% male plants to ensure adequate pollination and fruit set (Rasul et al., 2007). The crop thrives best when propagated through tubers or stem cuttings, with tuber-based crops yielding 40–50 q/ha, compared to 20–25 q/ha from seeds or stem cuttings in the first year (Tiwari et al., 2022b). For vegetative propagation using known male and female plants, approximately 2500–2650 planting units per hectare are required, ensuring a favourable female-to-male ratio (approx. 2200–2300 females and 300–350 males). The pollination in this entomophilous crop typically begins 50–60 days after transplanting or 30–40 days after tuber sprouting. Evening anthesis and dependence on insect pollinators make artificial pollination a viable strategy to improve fruit set and yield (Tiwari et al., 2022b).

Spine gourd is a perennial dioecious climber commonly known as kankoda or teasle gourd and is widely distributed in Bangladesh, China, India, Japan, and Pakistan, being indigenous to tropical regions of Africa, South America, and Asia; although the exact origin of the *Momordica* genus remains uncertain, several experts have suggested eastern Asia, particularly eastern India or southern China, as a likely centre of domestication (Nagarani et al., 2014). The crop exhibits significant nutritional value, ecological adaptability, and established traditional applications; however, it remains underutilized due to limited agronomic refinement, insufficient breeding and genetic improvement efforts, and poor integration into value chains and structured vegetable production sectors. Moreover, much of the knowledge regarding its medicinal potential is derived from traditional practices and dispersed experimental studies, which has restricted its recognition within established healthcare and pharmaceutical research frameworks, thereby creating a gap between traditional importance and broader scientific integration that has constrained wider agricultural and clinical utilization of the crop. Despite its recognized nutritional and therapeutic potential, spine gourd faces several barriers to commercialization and wider acceptance as a functional food, including its dioecious nature, poor seed viability, seed dormancy, and the absence of a structured seed production system, which complicate large-scale propagation and uniform crop establishment, while asynchronous flowering and dependence on effective pollination further limit yield reliability (Chatterjee et al., 2025).

In addition, the limited availability of improved cultivars, narrow genetic base of released varieties, and vulnerability to pests and diseases restrict consistent production and market standardization, and progress in genetic improvement has historically been slow due to biological constraints and comparatively limited agronomic research attention (Chatterjee et al., 2025). However, the genetic diversity of the species is under threat, particularly in forested and fallow lands where only female tubers are traditionally harvested, and this biased selection, combined with habitat disturbance and the widespread use of systemic non-selective herbicides, has led to reduced population vigour and loss of genetic variability in wild populations, making conservation efforts and sustainable cultivation practices urgently needed. Although conventional propagation methods continue to dominate, they face limitations, and *in vitro* propagation, already applied to other *Momordica* species, presents an efficient alternative for producing true-to-type planting material at scale; however, callus-mediated regeneration systems carry risks of soma clonal variation, highlighting the need for improved protocols using nodal explants (Rai et al., 2012).

Given the growing interest in spine gourd for its agronomic, nutritional, healthcare and economic traits, this review critically examines its taxonomy, Botany, Phytochemistry, current cultivation practices, reproductive biology, propagation constraints, and opportunities for harnessing its potential for the healing of various ailments Such an appraisal is timely to inform breeding programs, support conservation strategies, and promote sustainable intensification of this promising yet underutilized crop.

## Geographical distribution of spine gourd spp.

Spine gourd (*Momordica dioica*) is native to tropical and subtropical regions of Asia and exhibits strong ecological adaptability, thriving under a variety of environmental conditions. It flourishes in warm, humid climates with abundant sunlight and tolerates a wide range of soil textures, from sandy loams to heavier clay soils. This resilience enables its naturalization across diverse habitats, including forest undergrowth, shrublands, and anthropogenically disturbed areas such as field margins and roadsides (Das et al., 2022).

The species exhibits a broad geographical distribution in India, where it commonly occurs in semi-wild habitats across states such as Jharkhand, Chhattisgarh, and Odisha (Janani and Balusamy, 2018), as well as in forest-dominated regions of Assam and Tripura (Das et al., 2022). The tender, spiny green fruits (locally known as Kantola) are widely consumed during the monsoon season and are well integrated into regional food systems, while the crop is also cultivated across diverse agroecological zones including Maharashtra, Gujarat, West Bengal, Madhya Pradesh, Karnataka, Uttar Pradesh, Bihar, parts of southern India, and the Andaman Islands (Ram et al., 2002; Janani and Balusamy, 2018). Beyond India, cultivation and utilization of spine gourd geographically extend across several tropical regions of Asia, with wild populations reported in countries such as Japan and Malaysia, indicating the region as a likely centre of origin owing to the presence of both wild and cultivated forms (Ram et al., 2002). Indian populations display substantial genetic diversity; however, increasing deforestation and habitat disturbance have accelerated genetic erosion, prompting local domestication efforts in regions such as coastal Odisha and underscoring the need for urgent germplasm conservation and sustainable cultivation strategies (Ram et al., 2002).

## Species diversity

Within the genus Momordica, 47 species are native to Africa, while 12 species occur across Asia and Australia. All species bear unisexual flowers, though sexual expression varies regionally (Schaefer and Renner, 2010). African species are nearly evenly divided between dioecious and monoecious forms, whereas all Asian *Momordica* species are dioecious, indicating a continental shift in reproductive biology. In India, spine gourd is an important dietary component in rural and peri-urban regions, particularly in tribal and rainfed areas, where it is cultivated in home gardens or collected from forests during the rainy season. Consumers generally prefer fruits with uniform shape, moderate spines, and green coloration (Yadav et al., 2024).

Phylogenetic analyses indicate that *Momordica c*onstitutes a monophyletic group, suggesting a single ancestral origin (Schaefer and Renner, 2010). Evolutionary evidence further indicates that monoecy has arisen repeatedly from dioecy on at least seven independent occasions, predominantly among African species adapted to sparsely populated savanna environments, reflecting an early developmental transition toward the coexistence of both sexes on a single plant. The genus comprises 59 species distributed across diverse habitats including rainforests, deciduous forests, bushlands, savannas, and grasslands, with most species occurring as perennial climbers, alongside a few shrubs and annuals. Genetic data support a tropical African origin of *Momordica*, with subsequent long-distance dispersal into Asia approximately 19 million years ago (Schaefer and Renner, 2010). The name *Momordica* is derived from the Latin word *mordeo* (meaning “to bite”), referring to the characteristic grooved margins of the seeds. Among the species, *M. dioica* is particularly valued for its nutritional and medicinal attributes (Bharathi et al., 2014; Nagarani et al., 2014), and the native geographical distribution of major *Momordica* species is summarized in Table 1.

**TABLE 1:** Major Species and their origin under the *Momordica* genus.

S.no	Species	Native regions
1	*Momordica cissoides* Planch. ex Benth.	Central Africa
2	*Momordica anigosantha* Hook.f.	Eastern Africa and the Great Lakes Region
3	*Momordica spinosa* Chiov.	The Horn of Africa (East and South Ethiopia to Kenya)
4	*Momordica boivinii* Baill.	Eastern and Southern Africa
5	*Momordica calantha* Gilg	Eastern African Rift Valley including North Mozambique
6	*Momordica denudate* C.B.Clarke	Western India and Sri Lanka
7	*Momordica cochinchinensis* (Lour.) Spreng.	Tropical and Subtropical Asia and North Queensland
8	*Momordica cymbalaria* Hook.f.	North East Africa, Pakistan and India
9	*Momordica dioica* Roxb. ex Willd.	Indian Subcontinent to Myanmar
10	*Momordica charantia* L. - Bitter melon	Sub-Saharan Central Africa, India and North West Australia
11	*Momordica foetida* Schumach.	Central and South Africa
12	*Momordica balsamina* L.	Tropical and South Africa, South West Arabian Peninsula and Australia
13	*Momordica denticulata* Miq.	West Malesia
14	*Momordica laotica* Gagnep.	Indian Subcontinent to South China and West Malesia
15	*Momordica multiflora* Hook.f.	West Africa (specifically Ghana, Uganda, and Angola)

Data source: Royal Botanic Gardens Kew (2024); Plants of the World Online (2024a); Plants of the World Online (2024b) and International Plant Names Index (2024); distributional data cross-verified with GRIN (Germplasm Resources Information Network).

## Taxonomy

The genus *Momordica* belongs to subtribe *Thladianthinae,* tribe *Joliffieae,* subfamily *Cucurbitoideae,* of the *Cucurbitaceae* (Jeffrey, 1990). According to Robinson and Decker-Walters (1997), the genus has 45 species domesticated and cultivated across Asia and Africa. In India itself six species of the *Momordica* genus are predominantly cultivated. These species can be clustered under two headings: the monoecious group comprising *M. charantia* L. and M. *balsamina* L. and the dioecious group *includes M. dioica* Roxb., *M. sahyadrica* Joseph and *M. cochinchinensis* (Lour.) Spreng., and *M. subangulata* Blume (ssp. *renigem* (*G.* Don) W.J.J.de Wilde). The species *Momordica* cymbalaria Fenz. [also referred to as *Luffa* cymbalaria or M. tuberosa (Roxb.) Cognj] is not formally listed in the current classification. However, some researchers continue to regard it as part of the *Momordica* genus (Joshi et al., 2005). According to Plants of the World Online (POWO, Royal Botanic Gardens, Kew), the taxonomic classification of Momordica dioica Roxb. is as follows:

- Kingdom: Plantae
- Phylum: Streptophyta
- Class: Equisetopsida
- Subclass: Magnoliidae
- Order: Cucurbitales
- Family: Cucurbitaceae
- Genus: *Momordica*
- Species: *Momordica dioica* Roxb.

## Botany: monoecy/dioecy morphology

Spine gourd (*Momordica dioica*) is a perennial climbing plant with separate male and female individuals, classified under the family Cucurbitaceae. In geographical distribution the plant is characterized by slender, glabrous stems supported by simple tendrils, allowing it to climb over surrounding vegetation. Its leaves are alternate, broadly ovate, and deeply 3–5 lobed, with a cordate base, denticulate margins, and an acute apex (Bawara et al., 2010). These traits collectively contribute to a dense canopy structure and efficient photosynthetic performance. The plant bears tuberous roots, which support perennial regrowth and adaptability to varied soil and climatic conditions.

**FIGURE 1:**
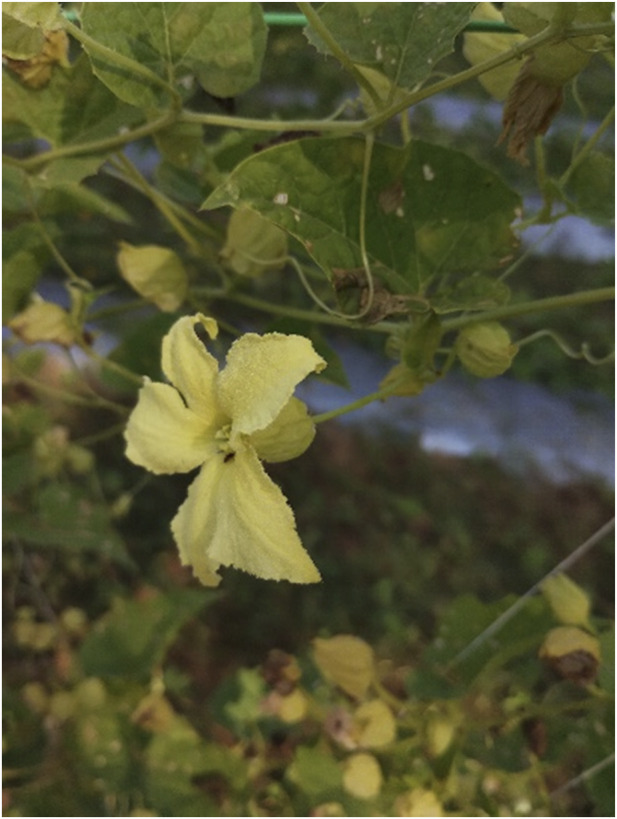
Male flower.

**FIGURE 2:**
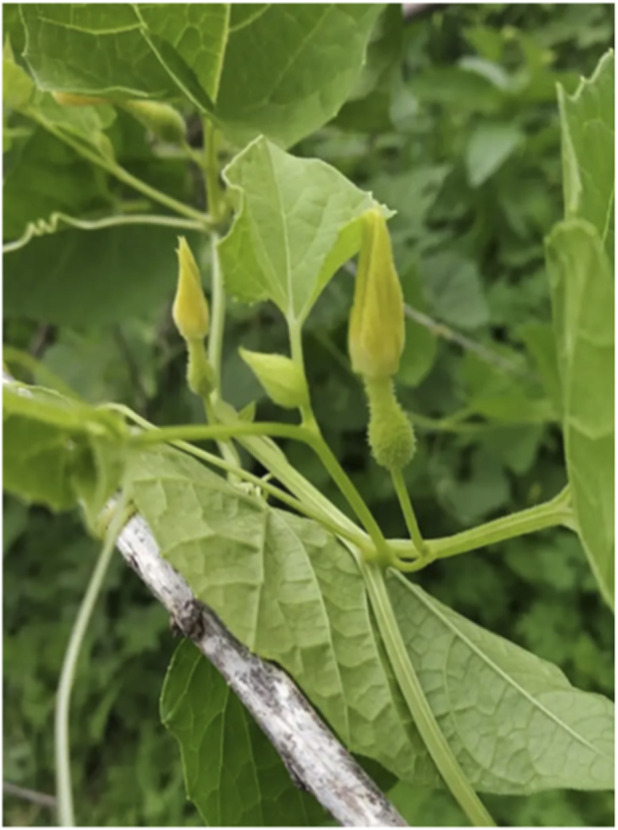
Female flower.

The species exhibits dioecy, with male and female flowers produced on separate plants. Both are solitary and borne in axillary positions. Male flowers measure approximately 2.8–3.5 cm, have five oblong petals and three stamens, while female flowers are slightly smaller, comprising three distinct nectar glands and a well-developed pistil (Bawara et al., 2010). Floral development is asynchronous during the early stages but becomes synchronized at anthesis, which typically occurs around 7:00 a.m. and lasts for about an hour. The fruits are ovoid to ellipsoid pepos (Asna et al., 2020), 2.5–6.5 cm in length, and densely covered with short, blunt spines. During ripening, they change from green to a bright yellow or orange hue. Internally, the fruit contains numerous seeds that are small (6–7 mm), slightly flattened, ridged, and range in colour from yellow to brown (Asna et al., 2020). Figures 1, 2 shows male and female flowers of spine gourd respectively.

**FIGURE 3:**
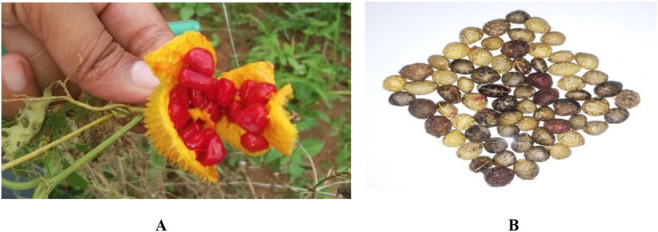
Spine gourd seeds. **(A)** Seeds in ripened spine gourd fruits. **(B)** Dried seeds of spine gourd.

*Momordica dioica* exhibits a dioecious reproductive system, with male and female flowers borne on separate plants and marked differences in floral phenology and morphology. Male flowers (staminate) mature earlier, attaining anthesis at approximately 4:00 a.m., with pollen dehiscence occurring around 6:00 a.m., whereas female flowers (pistillate) typically open later, near 6:00 a.m. Stigma receptivity begins nearly 12 h prior to anthesis and extends up to 18 h post-anthesis, reaching its maximum at the time of anthesis; as a result, effective fertilization under field conditions often requires manual pollination between 5:00 a.m. and 6:00 a.m. (Sandilya et al., 2019). Under natural environmental conditions, fruit set is reported to be approximately 22%, whereas controlled hand pollination can result in nearly 100% fruit set, highlighting the reproductive constraints associated with dioecy (Sandilya et al., 2019). Pollen grains are spherical, yellow, and tricolporate, exhibiting very high initial viability (≈98%), although viability declines with increasing temperature and humidity during storage, while sucrose or glucose solutions enhance pollen germination and pollen tube growth, indicating a nutritive role of carbohydrates. Considerable morphological variation is observed across the *Momordica* genus, particularly in reproductive traits (Sandilya et al., 2019). The seeds of mature fruits are enclosed in a characteristic red pulp (Figure 3A), and upon removal, the seeds exhibit coats ranging from brown to black (Figure 3B); comparative botanical differences among major cultivated *Momordica* species are summarized in Table 2.

**TABLE 2:** Botanical differences among the major cultivated *Momordica* species.

Species	Plant	Stem	Leaves	Flower	Fruit	Seeds	References
*Momordica charantia*	A monoecious much branched vigorous climbing annual	Angled, grooved, young parts densely hairy, older branches more or less pubescent	Almost orbicular or reniform in outline, lobes ovate-oblong, acute or subacute, apiculate	Monoecious, male flowers solitary, peduncles slender, glabrous or slightly pubescent; Corolla somewhat irregular, lemon yellow; Female flowers on 5–10 cm long slender peduncles, bracteate usually at or near the base	Bright orange coloured, 5–15 cm long, fusiform, ribbed, with numerous triangular tubercles giving it the appearance of crocodile skin	Compressed, oblong, sub-bidentate at base and apex, sculptured on sides, cream or grey coloured	Kumar and Bhowmik (2010)
*Momordica dioica*	A dioecious, perennial climber with a tuberous root	Slender, glabrous to rarely sparsely pubescent, angled and sulcate	Much variable, membranous, ovate, obtuse or acute and mucronate, lobes triangular	Male flowers solitary, glabrous peduncles which are hairy, Corolla yellow, Female flowers bracteate or ebracteate	Ellipsoid, shortly beaked, densely echinate with soft spines, apex shortly rostrate and annular, base usually rounded	Many, much variable in size and shape, turgid, more or less puriform, quite smooth	Chatterjee et al., 2023; Yadav et al., 2024
*Momordica cochinchinensis*	A dioecious, vigorous, perennial climber with a fibrous and tuberous root	Slender, sparsely pubescent, glabrous, angled and sulcate, sometimes tomentose at nodes	Simple, alternate, spiral, slightly yellow to brown, pubescent or glabrescent, cordate or broadly ovate-orbicular, cordate at base, acute at apex, undulate-dentate margin, lobes ovate or oblong, lanceolate	Monoecious, Male flowers solitary or in a short raceme; bracteate at apex; orbicular-reniform, entire, retuse at apex; calyx tube funnelform; broadly lanceolate or oblong, apex acute; corolla yellow; 3 stamens; Female flower solitary; bracteate at middle	Round or oblong, 13–20 cm long, small spines covering the exocarp, red, ovoid, fleshy, densely spinescent, apex rostellate. Upon ripening colour changes from green to yellow, orange and finally red when harvested	Present in clusters, thick, ash grey, ovoid or square, both surfaces sculptured, margin undulate-sub lobulate	Nguyen and Kha, 2022; Othman et al. (2020)
*Momordica balsamina*	A monoecious, much branched, climbing perennial from a tuberous root	Slender, glabrous	Herbaceous or slightly hairy particularly on nerves beneath, lobes rhomboid or obovate to elliptic-rhomboid	Monoecious, all solitary; Male flowers on slender, filiform peduncles, glabrous or somewhat hairy towards apex, corolla pale yellow; Female flowers on ebracteate or bracteate peduncles	Sub globose to ovoid, with a broad, conical rostrum, abruptly and shortly attenuate at base, bright orange-red to scarlet when ripe	With a carmine red arillus, grey, ovate or oblong in outline, compressed	Chinthan et al. (2022)

Recent work by Chatterjee et al. (2023) on 96 first-generation mutant lines of *M. dioica* revealed substantial morphological variability, indicating the success of mutation breeding in expanding the phenotypic base. Leaf length varied from 3.18 to 21.98 cm and leaf width from 2.43 to 11.44 cm, while pedicel length ranged between 1.74 and 9.21 cm. Ovary length and diameter were found to range from 16.40 to 31.60 mm and 3.80–8.00 mm, respectively. Style length extended from 3.60 to 8.00 mm, and pistil tip length ranged from 2.20 to 6.40 mm. Fruit traits also exhibited marked diversity: fruit length ranged from 3.62 to 7.92 cm, diameter from 2.82 to 5.38 cm, and individual fruit weight from 5.83 to 33.56 g. Such wide variation across vegetative and reproductive traits highlights the potential of mutation breeding for future genetic improvement, especially for optimizing plant architecture and yield components.

## Cultivation practices

**FIGURE 4:**
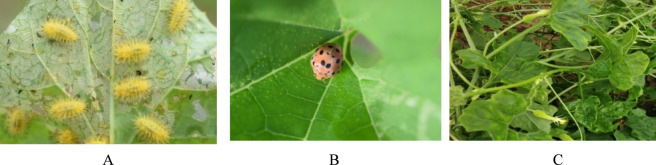
Various pest and diseases of spine gourd. **(A)** Epilachna beetle (Grub). **(B)** Epilachna beetle (Adult). **(C)** Viral infection.

The cultivation of spine gourd is dependent on the several factors as described below:

1. Adaptability: Spine gourds thrive in a wide range of climates, including warm arid, temperate, and subtropical zones (Lim, 1998; Tiwari et al., 2022b). Their day-neutral growth habit allows them to produce flowers and fruits regardless of day length variations.
2. Soil and Temperature: Sandy loam or clay soils with a pH between 5.5 and 7.0 are ideal for spine gourd cultivation. These soils offer good drainage and contain high levels of organic matter, both crucial for healthy plant development. The ideal temperature range for spine gourd cultivation falls between 27 °C and 32 °C (Bairwa and Rana, 2017).
3. Irrigation: During the Kharif season (monsoon season in South Asia), spine gourds typically don't require additional irrigation due to increased rainfall. However, supplemental watering is necessary during periods of drought or heat stress to maintain optimal growth. Maintaining adequate moisture levels in the top 50 cm of the soil is essential, as this is where most of the roots are concentrated (Tiwari et al., 2022b).
4. Weed Control Strategies: Effective weed control, particularly during the initial growth stages, is crucial for maximizing spine gourd productivity. Weeds compete with young plants for water, nutrients, and sunlight (Tiwari et al., 2022b). Weed management in spine gourd cultivation involves an initial weeding to enhance soil aeration and minimize competition for young plants, using either manual or mechanical methods. To maintain a weed-free field, two additional rounds of hand weeding are recommended at 30 and 40 days after transplanting. Mulching after the first irrigation further helps by suppressing weeds, preventing soil crusting, and improving aeration. While organic mulches like paddy straw or dry grass offer a cost-effective solution, plastic mulch with planting holes can also be utilized (Tiwari et al., 2022b).
5. Pest and disease management: Spine gourd cultivation is susceptible to attack from various insect pests, with the most concerning being the polyphagous fruit fly (*Bactrocera cucurbitae*). This versatile pest, capable of feeding on a wide range of gourd crops, poses a significant threat to spine gourd yields. Other notable insect pests include the Epilachna beetle (*Henosepilachna vigintioctopunctata*) and the Red pumpkin beetle (*Aulachophora foveicollis*). Figure 4 illustrates the common pests and diseases that challenge spine gourd, based on the observations of Tiwari et al. (2022b). While complete eradication of the fruit fly might not be achievable, a multi-pronged approach can effectively suppress their populations and minimize economic damage. This strategy could involve the application of specific chemical insecticides like chlorpyriphos (0.04%) as foliar sprays (Tiwari et al., 2022b). However, the use of chemical insecticides should be part of an Integrated Pest Management (IPM) program that incorporates alternative methods. These methods can include the use of poison baits that combine insecticides with female fruit fly sex attractants to lure and eliminate adult flies. Additionally, applying soil-based insecticides can target emerging fruit fly pupae before they mature and contribute to the pest population. Implementing these strategies in combination can promote a more sustainable approach to pest control in spine gourd cultivation (Angon et al., 2023).

## Propagation techniques

**FIGURE 5:**
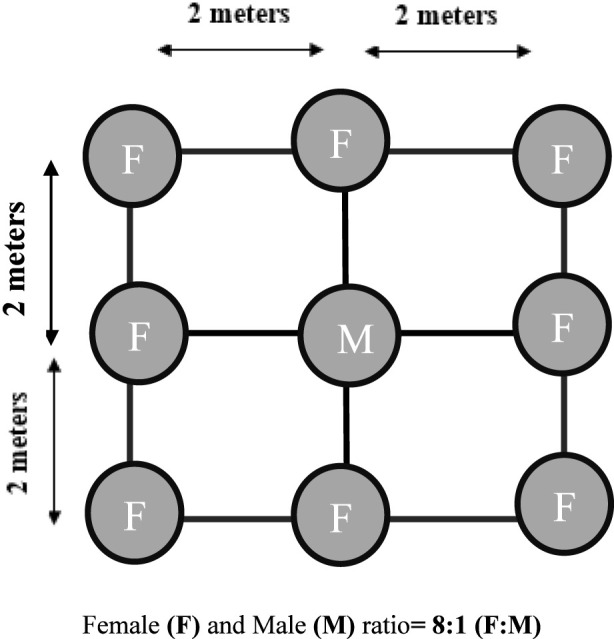
Planting model of spine gourd.

Spine gourd is commonly propagated using tubers, which are planted in pits measuring 30 × 30 × 30 cm, spaced at 2 × 2 m across the plot. Before planting, the pits are left open to sunlight for about a week to help reduce pest and disease presence. Once treated, they are filled with a mix of farmyard manure, NPK fertilizers, and insecticides such as chlorpyriphos, after which the tubers are carefully placed inside. To support healthy growth, nitrogen may be applied occasionally as a top dressing (Tiwari et al., 2022b). In addition to tubers, spine gourd can also be propagated through seeds or stem cuttings, with the same 2 × 2 m spacing maintained regardless of the method used (Tiwari et al., 2022b). Figure 5 shows the planting model of spine gourd as suggested by Tiwari et al., 2022b.

**FIGURE 6:**
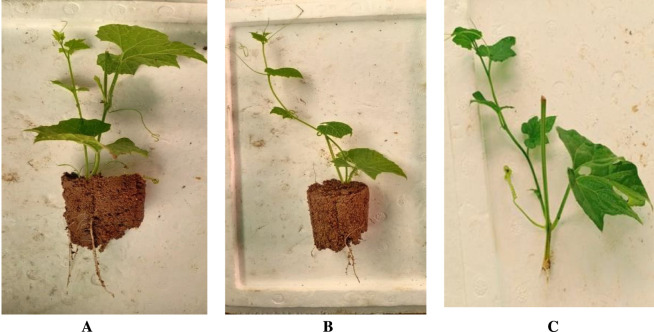
Growth observed in different types of stem cuttings. **(A)** Double node cutting. **(B)** Single node cutting. **(C)** Cutting without any treatment.

**FIGURE 7:**
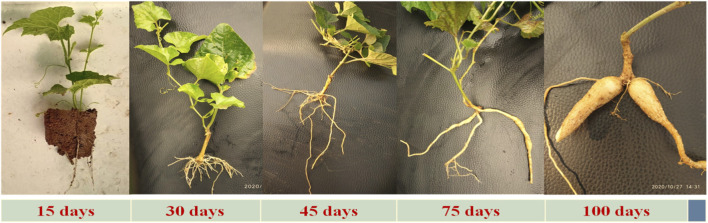
Growth of plant from stem cuttings at various day intervals till the tuber formation.

**FIGURE 8:**
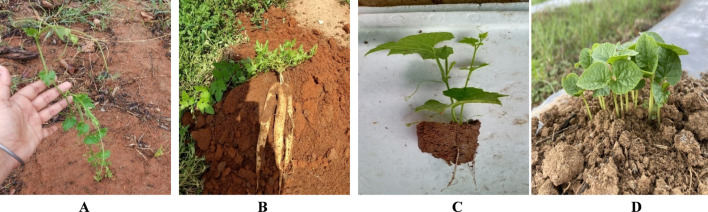
Plants obtained through various propagation methods. **(A)** Plant obtained through seed. **(B)** Plant obtained through tuber. **(C)** Plant obtained through stem cutting. **(D)** Plant obtained through micropropagation.

**FIGURE 9:**
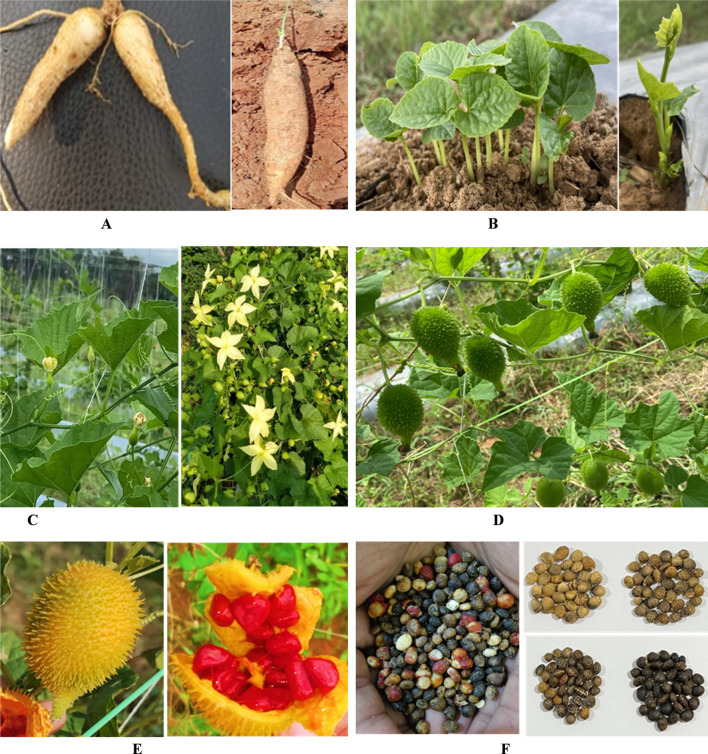
Various stages within the life cycle of spine gourd. **(A)** Tubers for planting. **(B)** Sprouting. **(C)** Flowering [Female (left) and Male (Right)]. **(D)** Fruiting. **(E)** Ripening. **(F)** Seeds.

Motapalukula et al., 2020, have mentioned are several methods of propagating spine gourd viz. through seeds, tubers, stem cuttings etc. Different propagation methods used in spine gourd differ markedly in their efficiency and applicability, each presenting specific advantages and constraints that influence its commercial cultivation. Propagation through seeds is limited by dormancy, inconsistent germination, and unpredictable sex expression, whereas tuber and stem-based methods are constrained by low multiplication rates and inconsistent establishment success. These constraints associated with individual propagation methods are summarized in Table 3. In addition, Figure 6, as shown by Tiwari et al. (2022a), illustrates the types of stem cuttings (double node, single node, and untreated cuttings), while Figure 7, from Tiwari et al. (2022b), shows the growth response of these propagated plants at 15, 30, 45, 75, and 100 days after planting, thereby highlighting the performance of the different propagation methods. Figures 8A–D shows spine gourd plants grown using different propagation methods viz. seed, tuber, stem cutting, and micropropagation. Figures 9A–F illustrates the various stages in the life cycle of the spine gourd.

**TABLE 3:** Propagation methods and their Key Limitations of Spine Gourd.

Propagation method	Description	Limitations	References
Seed Propagation	Propagation through seeds provides a basic method for establishing spine gourd plants. However, freshly harvested seeds exhibit dormancy and typically require 5–6 months of storage to overcome this physiological barrier. Pre sowing practices, such as soaking seeds in tap water for 24 h, have been shown to enhance germination rates. However, propagation through seeds presents two main challenges, it does not allow control over the male-to-female plant ratio, and it generally results in a longer period before fruiting when compared to other propagation methods.	Unpredictable Sex Ratio: Seed-sown populations naturally segregate into male and female plants, but sex cannot be determined at early stages. This necessitates thinning of excess male plants after flowering to maintain an ideal 10% male ratio for pollination, which leads to increased seed use, wasted resources, and higher input costs. Although molecular and biotechnological technologies have enabled early sex identification, they still remain unsuitable for widespread adoption by farmers. Low Germination: Low germination rates in spine gourd are largely due to the hard seed coat, which acts as a physical barrier. Treatments such as soaking or acid scarification using agents like sulfuric acid have been found to improve germination, though they also require additional handling and resources. Delayed Fruiting: Seed propagated plants generally need more time to sprout and reach the flowering stage, which delays the overall harvest schedule Reduced Seed Viability: Seed viability in spine gourd is often low, which makes seed based propagation even less reliable.	Tiwari et al., 2022b; Motapalukula et al., 2020
Propagation via Tuberous Roots	Using tubers rather than seeds eliminates dormancy related delays and often produces robust, vigorous plants. Tubers are collected from mature (2–3-year-old) plants and cut into segments weighing 80–120 g, ensuring each contains two viable buds. Planting is optimally carried out during two seasonal windows—September to October or early February to March—across the Asian subcontinent. A planting distance of 2–3 m is recommended to ensure sufficient space and aeration for optimal growth.	Limited Tuber Multiplication: Each mature spine gourd plant develops only one primary tuber and lacks secondary tuber production, making large-scale multiplication inefficient. Consequently, farmers need to procure a greater number of tubers, which can be both logistically and financially challenging. Increased Production Cost: The limited capacity for multiplication directly increases the cost of planting materials, making this method relatively expensive for commercial-scale cultivation.	Tiwari et al., 2022b; Motapalukula et al., 2020
Propagation via Stem Cuttings	This method involves the use of terminal shoot cuttings to propagate new plants. To promote rooting, the cuttings are treated with 1500 ppm Indole-3-butyric acid (IBA) and planted in a soil mixture prepared in a 1:2:1 ratio of soil, sand, and compost.	Low Success Rate: Vine or stem cuttings often show lower success in establishing mature, productive plants, which limits the popularity of this method. Hormonal Dependence: Root initiation depends heavily on the application of plant growth regulators such as IBA. This adds complexity, cost, and the need for technical know-how among growers.	Tiwari et al., 2022a; Motapalukula et al., 2020
Micropropagation (Tissue Culture)	Micropropagation offers a modern and precise approach for spine gourd propagation, particularly useful for commercial production. It enables the generation of genetically identical plants with a predefined sex (male or female), thus ensuring crop uniformity and eliminating the need for later thinning of male plants. Nodal explants containing actively growing axillary buds are cultured on Murashige and Skoog (MS) media supplemented with defined concentrations of auxins and cytokinins to induce shoot and root formation. This method is highly efficient for producing true-to-type plants from elite or selected lines.	Genetic Uniformity and Sex Control: Micropropagation ensures consistency in plant characteristics and allows sex pre-selection, which is particularly beneficial for fruit production. Explant Selection and Media Dependency: The success of this method hinges on the appropriate choice of explant material and the precise composition of the growth media, including hormone concentrations. Importance for Elite Line Propagation: This technique is ideal for maintaining and multiplying plants with superior traits. High Cost and Technical Constraints: Despite its advantages, micropropagation is relatively expensive and requires skilled personnel, controlled environments, and standardized protocols to achieve consistent results.	Motapalukula et al. (2020)

Supporting Structures for Climbing Plants.

**FIGURE 10:**
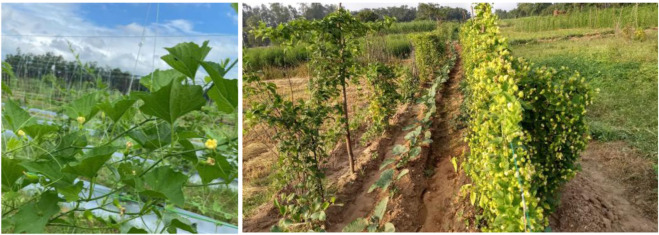
Stacking in spine gourd.

Since spine gourds are climbing vines, they require support structures for proper growth and development. Bamboo stakes measuring 2.2 m long and 25–30 cm wide are commonly used. These stakes are inserted near the plants and loosely tied to them at several points with rope as the vine grows. Stacking in spine gourd is shown in Figure 10.

## Harvesting

**FIGURE 11:**
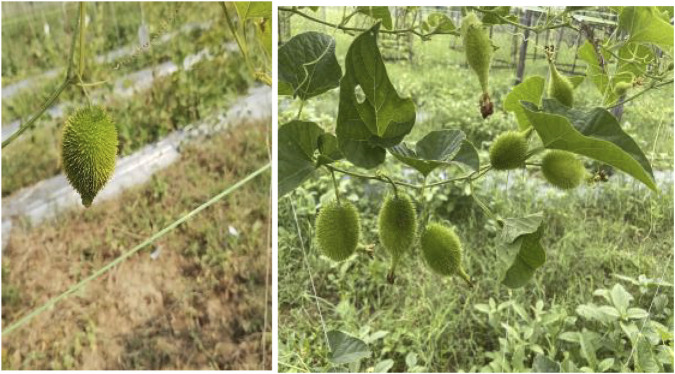
Matured fruits.

**FIGURE 12:**
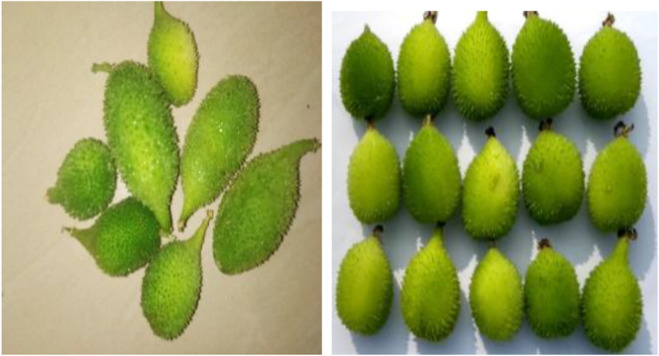
Harvested fruits.

Spine gourd harvesting requires careful attention to ensure fruit quality and marketability. Here’s a breakdown of the key practices involved (Tiwari et al., 2022b):

- Maturity Indicators: Spine gourds are harvested based on their horticultural maturity, considering factors like size, colour, and age. Specific criteria for these parameters will vary depending on intended consumption purposes. Figure 11 shows horticulturally matured fruits.
- Frequent Picking Schedule: Due to the rapid growth rate of spine gourds, fruits can quickly become unsuitable for the market if left unharvested. Therefore, frequent picking is essential throughout the harvesting period to maintain fruit quality and marketability.
- Manual Harvesting with Care: Spine gourds are typically hand-picked to minimize damage. During harvest, growers must exercise utmost care to avoid injuring the vine or the fruits themselves. This ensures the continued health of the plant and prevents spoilage of harvested fruits. Figure 12 shows harvested fruits.

## Nutritional composition of spine gourd

*Momordica dioica* Roxb. is a nutritionally valuable plant containing various essential elements. On a dry weight basis, 100 g of edible spine gourd provides a significant amount of nutrients as mentioned in Table 4.

**TABLE 4:** Nutritional composition of *Momordica dioica*.

Category	Metabolite	Amount/Composition
Proximate Composition	Moisture	84.1%
	Energy	311–325 kcal per 100 g
	Carbohydrates	45%–47.92%
	Protein	18%–19.38%
	Crude Fibre	21.3%–22%
	Lipids/Crude Fat	4%–4.7%
	Ash	6%–6.7%
Minerals	Calcium	33–35 mg/100 g
	Phosphorus	42–45 mg/100 g
	Iron	4–5 mg/100 g
	Sodium	1.51 mg/100 g
	Potassium	8.25 mg/100 g
	Magnesium	Present (trace–moderate levels)
	Zinc and Manganese	Trace amounts
Vitamins	Vitamin A (β-carotene)	2.5 g/100 g equivalent
	Vitamin B1 (Thiamine)	1.8 g/100 g
	Vitamin B2 (Riboflavin)	3.5 g/100 g
	Vitamin B3 (Niacin)	1.9 g/100 g
	Vitamin B5 (Pantothenic acid)	18 g/100 g
	Vitamin B6 (Pyridoxine)	4.3 g/100 g
	Vitamin B9 (Folic acid)	3.6 g/100 g
	Vitamin B12	4 g/100 g
	Vitamin D2 and D3	3 g/100 g
	Biotin (Vitamin H)	6.5 g/100 g
	Vitamin K	15 g/100 g
	Myristic acid	3.589%
Fatty Acid Profile (% of total fatty acids)	Palmitic acid	12.157%
	Stearic acid	3.547%
	Oleic acid	56.253%
	Linoleic acid	22.511%
	Alpha-linolenic acid	1.943%

Source: Tiwari et al., 2022b; Jha et al., 2017.

## Phytochemistry of spine gourd

### Bioactive compounds

Spine gourd is recognized for its rich phytochemical profile, which is believed to contribute to its potential medicinal and health-promoting properties. A number of these bioactive substances have been identified and studied for their health-related effects. According to Talukdar and Hossain (2014), some of the key bioactive metabolites identified in spine gourd that may contribute to its health-promoting effects include the following:

Triterpenoids: Spine gourd contains a range of triterpenoids, including momordin, momordicosides, and charantins. These metabolites have drawn interest for their potential to help manage blood sugar levels, ease inflammation, and possibly slow the growth of certain cancer cells.

Flavonoids: The plant is also a source of flavonoids such as quercetin, kaempferol, and rutin. These natural antioxidants are known to help the body cope with oxidative stress and may play a role in calming inflammation and supporting overall immune health.

Sterols: Plant sterols like stigmasterol and β-sitosterol are also present in spine gourd. Research suggests that these sterols might offer various health benefits, including potential cholesterol-lowering effects and anti-cancer properties.

Saponins: *Momordica dioica* Roxb. is known to contain saponins, a class of metabolites with antimicrobial and immunosuppressive properties. This presence of saponins could potentially explain the historical use of spine gourd in traditional medicine for treating infections and potentially enhancing immune function.

Alkaloids: Spine gourd contains alkaloids, including momordine and momordicinine, which have exhibited promising anti-diabetic effects. Research suggests a potential link between these alkaloids and glucose metabolism (Talukdar and Hossain, 2014). It has been reported that alkaloids can act as anti-diabetic agents by inhibiting enzymes involved in carbohydrate digestion, thereby reducing glucose absorption, and by stimulating insulin secretion or enhancing insulin sensitivity, leading to improved glucose uptake by cells. Alkaloids modulate glucose metabolism through the regulation of multiple protein targets, including AMP-activated protein kinase, glucose transporters, glycogen synthase kinase-3, sterol regulatory element-binding proteins, glucokinase, glucose-6-phosphatase, and acetyl-CoA carboxylase, exerting their effects via either inhibitory or stimulatory mechanisms (Muhammad et al., 2021).

Phenolic compounds: Spine gourd’s phenolic profile includes catechins, epicatechins, and gallic acid derivatives. These classes of phenolics are well-known for their antioxidant properties (Talukdar and Hossain, 2014). These molecules function as electron donors, effectively neutralizing harmful free radicals. Their hydroxyl groups readily donate hydrogen atoms to stabilize reactive oxygen species (ROS), thereby preventing oxidative damage to cellular components. These metabolites are instrumental in safeguarding cellular integrity, mitigating chronic diseases, and promoting overall well-being. Some researchers have reported the potent antioxidant activity of aqueous extracts of spine gourd (Sharma and Singh, 2014). In another study, oral administration of *Momordica dioica* fruit pulp protein extract to streptozotocin-induced diabetic rats for 30 days significantly ameliorated hyperglycemia, impaired glucose tolerance, and weight loss. Furthermore, the extract exhibited hypolipidemic, hepatoprotective, and renoprotective effects, as evidenced by improved antioxidant status (Shreedhara and Vaidya, 2006; Nadkarni and Nadkarni, 2007; Publication and Information Directorate, 1962; Poovitha et al., 2017).

The presence of these bioactive metabolites in spine gourd highlights its potential as a valuable source of natural antioxidants, anti-inflammatory agents, and metabolites with potential therapeutic applications. Table 6 contains the phytochemical metabolites in various plant parts of spine gourd. Based on existing studies, spine gourd is amenable to utilization in multiple forms, including fresh or processed foods, dried powders, fortified food products, and experimental extracts for pharmacological evaluation. However, pharmaceutical formulations would require further standardization, safety validation, and clinical assessment. At present, standardized therapeutic doses and validated preparations of spine gourd have not been established, as existing studies largely employ diverse experimental extracts under controlled conditions. Consequently, its health benefits are best interpreted in the context of traditional dietary use and functional food applications rather than defined therapeutic dosing.

### Extraction of compounds

The choice of solvent significantly influences the extraction process and subsequent antimicrobial activity of plant extracts:

Polar solvents like water and ethanol excel at extracting hydrophilic metabolites such as flavonoids, glycosides, and alkaloids. However, they might be less efficient in extracting lipophilic metabolites (Kumar et al., 2023). Non-polar solvents such as hexane and chloroform are adept at extracting lipophilic metabolites like terpenoids and essential oils. However, they may not effectively extract polar compounds (Kumar et al., 2023).

Methanol is a versatile solvent capable of extracting both polar and non-polar compounds, making it a popular choice for initial screening. Selecting the most effective solvent for extracting compounds from a given plant typically needs careful adjustment to achieve the best results (Kumar et al., 2023).

The extraction method significantly influences the yield and composition of plant extracts, consequently impacting their analgesic potential. Maceration and percolation offer relatively simple approaches but often yield lower extraction efficiencies compared to Soxhlet extraction and ultrasonic-assisted extraction, which enhance solvent penetration and extraction rates. The choice of method directly impacts the spectrum of compounds extracted, with polar solvents favouring hydrophilic molecules and non-polar solvents targeting lipophilic constituents. This differential extraction can influence the presence of metabolites with analgesic properties, such as terpenes, flavonoids, and alkaloids. Moreover, the extraction process can affect the stability of bioactive metabolites, with factors like temperature and exposure time impacting their degradation (Basilio-Cortes et al., 2023). Consequently, optimizing the extraction method is crucial for maximizing the yield and bioactivity of analgesic compounds from plant matrices. Table 5 provides additional details on the traditional medicinal uses of various *Momordica dioica* plant parts and the researchers who documented them.

**TABLE 5:** Nutrient and phytochemical study of *Momordica dioica*.

Plant part	Classification	Metabolite	References
Fruit	Crude protein Crude lipid Crude fiber Calcium	—	Aberoumand (2011)
	Fat Protein	—	Singh et al., 2008; Aberoumand and Deokule, 2008
	Carbohydrate	—	Aberoumand, 2011; Singh et al., 2008
	Potassium Sodium Iron	—	Aberoumand, 2011; Aberoumand and Deokule, 2008
	Thiamin Niacin	—	Singh et al. (2008)
	Carotene	—	Singh et al. (2008)
	Ascorbic acid	—	
	Zinc	—	Aberoumand, 2011; Aberoumand and Deokule, 2008; Tirmizi et al., 2007
	Chromium	—	Ghosh (2005)
	Iodine	—	Bhuiya et al. (1977)
	Phytic acid Total phenolic compound	—	Aberoumand and Deokule, 2008; Tanneeru et al., 2024
	Alkaloids Flavonoid Steroids Saponins Triterpenoid	—	Kumara and Bulugahapitiya, 2004; Tanneeru et al., 2024
	Alkaloid	Momordicin	Jian et al., 2005; Tanneeru et al., 2024
Seeds	Lectin	Anti-H-Lectin	Joshi et al. (2005)
	Alkaloid	—	Jian et al. (2005)
Roots	Stearic acid	—	Luo et al. (1998)
	Steroid	*α*-spinasterol-3-O-*β*-D-glucopyranoside	Luo et al. (1998)
		*α*-spinasteroloctadecanoate	Luo et al. (1998)
		Gypsogenin	Lei and Zuqiang (1997)
		Oleanolic acid	Lei and Zuqiang (1997)
		Hederagenin	Lei and Zuqiang (1997)
		3-O-*β*-D-glucopyranosyl gypsogenin	Luo et al. (1998)
		3*β*-O-benzoyl-11-oxo-ursolic acid	Lei and Zuqiang (1997)
		3-O-*β*-D-glucuronopyranosyl gypsogenin	Luo et al. (1998)
		3*β*-O-benzoyl-6-oxo-ursolic acid	Lei and Zuqiang (1997)
		3-O-*β*-D-glucopyranosyl hederagenin	Luo et al. (1998)

Source: Talukdar and Hossain, 2014; Jha et al., 2017; Venkateshwarlu et al., 2017.

The classification of metabolites in spine gourd as bioactive is supported by various scientific studies. Triterpenoids like momordin and momordicosides have demonstrated anti-diabetic, anti-inflammatory, and anti-cancer activities (Talukdar and Hossain, 2014). Flavonoids such as quercetin and kaempferol are well-documented for their antioxidant and anti-inflammatory effects (Talukdar and Hossain, 2014). Sterols including stigmasterol and β-sitosterol are linked to potential cholesterol-lowering and anti-cancer properties. Saponins are recognized for their antimicrobial and immunosuppressive properties, aligning with traditional uses for treating infections and enhancing immunity (Talukdar and Hossain, 2014). Alkaloids like momordine have shown promise in glucose metabolism regulation and anti-diabetic effects (Muhammad et al., 2021). Research has shown that spine gourd contains phenolic metabolites such as catechins and epicatechins, both of which exhibit strong antioxidant activity (Talukdar and Hossain, 2014; Sharma and Singh, 2014). These observations are well supported by scientific studies.

### Traditional uses and ethnobotanical of spine gourd

Spine gourd has been an integral part of traditional life in many indigenous communities, valued both as a food and a natural remedy. Once a wild-growing vine, it was gradually brought into cultivation, leading to a better understanding of its health benefits. Researchers have documented several traditional uses (Table 6), including its juice for managing high blood pressure (Kirtikar and Basu, 1999; Nadkarni and Nadkarni, 2007; Publication and Information Directorate, 1962), and cooked fruit as part of dietary care for diabetes (Kirtikar and Basu, 1999). It is also consumed fresh for its diuretic, liver-supporting, anti-inflammatory, and asthma-relieving properties (Nadkarni and Nadkarni, 2007; Publication and Information Directorate, 1962). Rubbing tender fruits on the skin is also noted for treating pimples and acne (Sharma and Joshi, 2004). The root is consumed as juice for diabetes (Kirtikar and Basu, 1999; Satyavati et al., 1976), used as an antitoxic agent for snake bites and scorpion stings (Kirtikar and Basu, 1934), and applied in powdered form for skin softening, reducing perspiration, and treating fever with delirium (Sharma and Joshi, 2004; Satyavati et al., 1976; Anjaria et al., 2002). Toasted roots are used to address bleeding piles and bowel infections (Jadeja et al., 2006). The mucilaginous tubers of the female plant are applied for bleeding piles and bowel infections (Jadeja et al., 2006). Roasted seeds benefit skin conditions such as eczema (Sharma and Joshi, 2004; Nadkarni and Nadkarni, 2007; Publication and Information Directorate, 1962), and leaf juice, when blended with other ingredients, forms an analgesic ointment (Kirtikar and Basu, 1999). Additionally, the traditional remedy ‘Panchatiktaghrita’, which includes spine gourd leaves, is used for chronic skin diseases (Sharma and Joshi, 2004; Sharma, 2004). This integrated perspective showcases the wide range of traditional uses and therapeutic applications of spine gourd across different plant parts.

**TABLE 6:** Ethnobotanical use of *Momordica dioica*.

Plant part	Mode of use	Ethnobotanical use	References
Fruit	Juice, cooked	Hypertension, diabetes, skin conditions, diuretic, laxative, hepatoprotective, anti-inflammatory, antipyretic, anti-asthmatic, anti-leprosy	(Kirtikar and Basu, 1999; Nadkarni and Nadkarni, 2007; Anjana et al., 2019; Publication and Information Directorate, 1962; Sharma and Joshi, 2004; Jha et al., 2017)
Root	Juice, powder	Diabetes, antitoxic, skin softening, antiperspirant, antipyretic, anti-hemorrhoidal, bowel infection reducer	(Kirtikar and Basu, 1999; Sharma and Joshi, 2004; Satyavati et al., 1976; Kirtikar and Basu, 1934; Anjaria et al., 2002; Jadeja et al., 2006; Jha et al., 2017; Kumar et al., 2017)
Mucilaginous Tuber	Paste	Anti-hemorrhoidal, bowel infection reducer	(Jadeja et al., 2006; Jha et al., 2017; Talukdar and Hossain, 2014)
Seed	Roasted	Eczema protectant, anti-hemorrhoidal	(Nadkarni and Nadkarni, 2007; Publication and Information Directorate, 1962; Sharma and Joshi, 2004; Jha et al., 2017; Talukdar and Hossain, 2014)
Leaf	Topical ointment	Skin disease reducer, analgesic, anti-bronchitic	(Kirtikar and Basu, 1999; Sharma and Joshi, 2004; Sharma, 2004; Jha et al., 2017)

The therapeutic claims associated with *Momordica dioica* are strongly plant part–specific and are mediated by distinct classes of bioactive metabolites. As summarized in Tables 5, 6, fruits, leaves, roots, seeds, and tubers are used for different ethnomedicinal applications, each supported by corresponding phytochemical profiles reported in experimental and literature-based studies. These data clearly indicate that no single plant part or metabolite accounts for all reported bioactivities, and that distinct organs contribute to different pharmacological effects.

### Culinary uses of spine gourd

Spine gourd has been part of traditional diets for generations. This versatile vegetable appears in a wide range of regional recipes. The young fruits, tender shoots, and leaves are commonly prepared in stir-fries, curries, pickles, and soups. The leaves are especially appreciated for their unique taste when cooked as a leafy green. Found growing naturally in the Western Ghats and coastal areas of India, spine gourd has long been part of local food traditions (Joseph et al., 2020). The immature fruits are widely used in everyday meals and often serving as a versatile ingredient in traditional meals. The tuberous roots can also be cooked and served as a vegetable, while the seeds yield a semi-drying oil that adds to the culinary applications.

In traditional Indian cooking, spine gourd holds a special place. It is used in dishes like Kheksa Aloo (a curry with potatoes) and Bharwan Karela/Kankoda (stuffed spine gourd). In South India, it features in preparations such as Thoran, Theeyal, and Kootucurry (Joseph John and Antony, 2006), highlighting its importance in diverse regional cuisines.

### Medicinal/healthcare uses of spine gourd

Spine gourd has a long history of use in traditional medical systems, including Ayurveda, where it has been employed for the management of various health conditions. It has been particularly associated with applications related to metabolic regulation, inflammatory disorders, and general health maintenance. The medicinal uses outlined in the following subsections are derived primarily from studies investigating specific plant parts, such as fruits, leaves, and roots, prepared in different traditional and experimental forms. Where appropriate, findings from other *Momordica* species are referred to for comparative context. The sections below summarize the reported medicinal and healthcare uses of spine gourd as documented in traditional practices and evaluated in experimental studies:

Digestive health: Spine gourd has traditionally been used to ease constipation and support digestion. While the exact mechanism is not fully understood, its fiber content is likely a key factor. The fruit contains about 3 g of dietary fiber per 100 g (Singh et al., 2008), which may help improve gut health by supporting beneficial gut bacteria. Its insoluble fiber also adds bulk to stools, promoting regular bowel movements and better digestive function.

Skin conditions: In many communities, spine gourd is applied externally to treat skin problems like eczema, acne, and fungal infections. Juice from unripe fruits is commonly used for acne and pimples, while roasted seeds are applied for eczema and other skin irritations (Nadkarni and Nadkarni, 2007; Publication and Information Directorate, 1962; Sharma and Joshi, 2004; Jatale et al., 2024). Though widely used, these remedies still need scientific validation.

Respiratory health: The plant is also known in folk medicine for helping with coughs, asthma, and bronchitis. While its exact role in easing respiratory symptoms isn't yet confirmed, traditional use suggests it may have broncho dilatory properties. Along with its digestive and liver-protective effects, spine gourd continues to be valued for its broad therapeutic potential (Jatale et al., 2024).

Anti-inflammatory effects: Traditional medicine records show that spine gourd has long been used to relieve inflammation, especially in cases of arthritis and swollen joints. Spine gourd exhibits potent anti-inflammatory, antioxidant, and antimicrobial properties, contributing to its immune-modulatory effects. These properties enable the plant to enhance immune function by supporting microbial defence, memory responses, and the activity of natural killer cells (Jatale et al., 2024). The antioxidant activity of spine gourd has been quite studied by the researchers (Hamsa et al., 2021; Publication and Information Directorate, 1962; Sharma and Joshi, 2004).

### Antioxidant activity

*Momordica* species are rich in carotenoids, endogenous antioxidants crucial for photosynthesis. These carotenoids are hypothesized to protect humans from carcinogens and oxidative stress linked to cardiovascular disease. Natural antioxidants, such as phenolics and polyphenolics abundant in bitter gourd, offer alternatives to synthetic antioxidants in preventing fruit deterioration. Bitter gourd fruits contain varying carotenoid profiles based on maturity, with cryptoxanthin as the primary pigment (Rodriguez et al., 1976). Additionally, these gourds also possess vitamin C, vitamin B complex, phenolic compounds, organosulfur compounds, and carotenoids (e.g., 3-carotene, zeaxanthin, lycopene, lutein, α-carotene) (Jayasooriya et al., 2000; Balentine et al., 1997; Wang and Mazza, 2002). Plant phenolics, in particular, show promise as dietary antioxidants by potentially reducing cholesterol, triglycerides, blood pressure, and cancer risk, contributing to cardiovascular health.

Also, in a comprehensive toxicity profile study of a saponin isolated from *Momordica dioica* fruit which was established in accordance with OECD guidelines, some toxic effects were found on the rats. Acute, sub-acute, sub-chronic, and chronic toxicity studies were conducted in rats. A single oral dose of 5,000 mg/kg body weight demonstrated no acute toxicity. Sub-acute studies at doses of 1000, 500, and 250 mg/kg body weight revealed no mortality or significant adverse effects at lower doses, while the highest dose induced mild toxicity. Sub-chronic and chronic studies at 500, 250, and 100 mg/kg body weight exhibited no observable clinical, haematological, biochemical, or histopathological abnormalities. These findings suggest that the saponin from *M. dioica* is well-tolerated at doses of 250 and 100 mg/kg body weight, with no evidence of toxicity after 180 days of administration (Jha et al., 2019).

### Anti-inflammatory activity

Scientific studies have explored the anti-inflammatory properties of *Momordica dioica* Roxb. (spine gourd). Shreedhara and Vaidya investigated the effectiveness of an alcoholic extract from the plant’s roots against liver damage induced by CCl_4_, demonstrating its potential anti-inflammatory properties. Similarly, another evaluation suggested the anti-inflammatory activity mediated by both hexane and methanolic extracts of the fruit pulp, further suggesting potential health benefits (Ilango et al., 2003).

### Anti-diabetic activity

*Momordica dioica* (spine gourd) is increasingly recognized for its potential role in diabetes management. Traditionally used in various regions, it is thought to help reduce blood glucose levels and improve insulin sensitivity. Extracts with appreciable amylase-inhibitory activity have been associated with improved glycemic control (Patel and Ishnava, 2015), and its anti-diabetic, anti-lipidemic, and antioxidant effects have been reported in several studies (Sharma and Singh, 2014; Poovitha et al., 2017). Although more clinical research is needed, findings from related species such as *M. charantia* (bitter gourd) lend support to its therapeutic potential. For instance, Ahmed et al. (1998) observed increased pancreatic β-cell regeneration in diabetic rats treated with bitter gourd juice, and Rathi et al. (2002). Robinson noted minimal toxicity in human subjects, suggesting a favourable safety profile. These findings indicate that *M. dioica* may offer similar benefits, warranting further investigation.

Multiple studies support the potential anti-diabetic properties of *Momordica dioica* Roxb. (spine gourd). Hamsa et al. investigated the effect of Khakra (a flatbread) supplemented with spine gourd powder on serum glucose levels in streptozotocin-induced diabetic rats. Their findings demonstrated a significant reduction in serum glucose levels (G6) at all measured intervals (0th, 7th, and 21st days) in the group receiving Khakra with spine gourd powder (10 g/rat/day) compared to the control group (Hamsa et al., 2021). Specifically, the G6 values were 199.48 mg/dL, 176.10 mg/dL, and 118.45 mg/dL for the LD + STZ + spine gourd Khakra group at the respective time points. The authors suggest that this hypoglycemic effect might be attributed to the activation of pancreatic β-cells and normalization of granulation, leading to increased insulin production (Kedar and Chakrabarti, 1982).

Pancreatic β-cells are the exclusive site of insulin biosynthesis and secretion in mammals. These cells function as glucose sensors, regulating insulin output in accordance with circulating glucose levels. Impaired insulin secretion underlies the hyperglycemia characteristic of type 2 diabetes, a global health crisis affecting over 450 million individuals. Glucose stimulation triggers both insulin secretion and augmented proinsulin biosynthesis via transcriptional and translational mechanisms (Rorsman and Ashcroft, 2018). Insulin biosynthesis and secretion in pancreatic β-cells is a complex process. The insulin gene is transcribed into mRNA and subsequently translated into the preproinsulin precursor. Within the endoplasmic reticulum (ER), this precursor undergoes cleavage to form proinsulin, which is further processed and folded. Proinsulin is transported to the Golgi apparatus where it is packaged into secretory granules and cleaved into mature insulin, C-peptide, and amylin. Upon calcium influx, these granules undergo exocytosis, releasing insulin and C-peptide into the bloodstream (Fu et al., 2013).

Furthermore, reported that aqueous extracts of *M. dioica* were more effective than the standard drug glibenclamide in lowering blood glucose levels in alloxan-induced diabetic rats, achieving a decrease of up to 76.90% (Singh et al., 2011). Similarly, it observed a marked decrease in serum glucose levels and a corresponding increase in serum insulin levels in streptozotocin-treated diabetic rats administered *Momordica dioica* methanolic extract (MDMtE) (Gupta et al., 2011).

### Reducing bad cholesterol

Several *Momordica* species, including *M. dioica*, *M. charantia*, and *M. cymbalaria*, have been studied for their potential role in supporting heart health by helping lower LDL cholesterol, a major risk factor for cardiovascular disease. *M. dioica*, in particular, appears to support insulin release and improve fat metabolism. Insulin helps control fat breakdown by reducing the activity of enzymes in fat tissue, which may in turn lead to lower cholesterol levels (Rauter et al., 2009).

This effect is also linked to the presence of natural metabolites like flavonoids, phenols, steroids, and saponins in these plants. Flavonoids and phenols act as antioxidants, protecting cholesterol from oxidative damage and supporting its removal from the body (Rauter et al., 2009; Marrugat et al., 2004). Plant-based steroids can reduce cholesterol absorption in the gut, while saponins bind to cholesterol and help prevent it from being absorbed. These compounds also encourage bile acid excretion, adding to their cholesterol-lowering potential (Harwood et al., 1993).

Supporting studies on animals have also been conducted. Hamsa et al. investigated the effects of spine gourd khakra on diabetic rats. Their findings showed a significant reduction in serum cholesterol levels in rats fed with spine gourd compared to the control group. The maximum reduction (39.63%) was observed in the group receiving the highest dosage (10 g/rat/day).

The flavonoids, phenols, steroids, and saponins in the gourd has complex beneficial effects on the overall functioning of glucose and fat metabolism viz insulin, plays a pivotal role in regulating glucose and lipid metabolism. By facilitating glucose uptake and promoting lipogenesis, insulin indirectly influences cholesterol levels through its association with weight management. Insulin resistance, a state of decreased cellular responsiveness to insulin, is linked to metabolic disturbances including dyslipidemia, often characterized by elevated cholesterol levels. However, the complex interplay between insulin, glucose, and lipid metabolism underscores the multifaceted nature of cholesterol regulation, with dietary, genetic, and lifestyle factors also exerting significant influence (Vargas et al., 2018).

### Nephroprotective activity

The *Momordica* genus shows particular promise for kidney health. Along with scientific researches traditional Ayurvedic medicine has also highlighted its potential for dissolving kidney stones. A 2011 study investigated the effects of *Momordica dioica* on kidney function in diabetic rats. They observed severe kidney degeneration in the diabetic control group. However, rats treated with *M. dioica* extract (MDMtE) or the standard diabetic medication glibenclamide displayed significant improvement in kidney histology. Microscopic examination revealed well-rejuvenated kidney structures in MDMtE-treated rats, including glomeruli, Bowman’s capsules, and proximal and distal convoluted tubules. These findings suggest that *M. dioica* may offer reno-protective (kidney-protecting) benefits in diabetes (Gupta et al., 2011). The researchers proposed two possible mechanisms: Renal Cell Regeneration: MDMtE might promote regeneration of kidney cells by enhancing protein synthesis or accelerating detoxification processes (Gupta et al., 2011).

*M. dioica* may also help reduce the harmful effects of free radicals and other damaging molecules like peroxynitrite. This antioxidant activity may play a role in supporting kidney health by helping to reduce lipid peroxidation (LPO) and limiting the production of nitric oxide (NO), both of which are linked to cellular damage (Gupta et al., 2011). MDMtE was found to help preserve kidney function in a diabetic rat model. Histological analysis indicated clear signs of renal damage in untreated diabetic rats, whereas those treated with MDMtE exhibited marked improvements in kidney tissue structure. The extract also contributed to lower blood glucose levels, highlighting its potential antidiabetic effect. It further supported kidney health by improving important biochemical indicators such as serum urea nitrogen, uric acid, and creatinine (Gupta et al., 2011).

The study also reported increased lipid peroxidation and reduced activity of antioxidant enzymes in the kidneys of diabetic rats, indicating oxidative stress as a key contributor to tissue damage. Elevated levels of lipid peroxidation and nitric oxide are known to cause cellular and membrane injury, leading to inflammation and functional decline. MDMtE appeared to counter these effects by restoring antioxidant balance and regulating nitric oxide levels, helping to reduce oxidative damage and maintain renal function. These findings suggest that its antioxidant action plays a central role in supporting kidney health in diabetic conditions (Gupta et al., 2011).

### Anticancer activity

Luo et al. (1998) investigated the anticancer potential of *Momordica dioica* root extracts by isolating bioactive constituents using chloroform (CHCl_3_). Three triterpenes and two steroidal compounds viz. α-spinasterol octadecanonate (I), α-spinasterol-3-O-β-D-glucopyranoside (II), 3-O-β-D-glucuronopyranosyl gypsogenin (III), 3-O-β-D-glucopyranosyl gypsogenin (IV), and 3-O-β-D-glucopyranosyl hederagenin (V); were characterized using spectral analyses (MS, IR, ^1^H-NMR, ^13^C-NMR, and DEPT). Both the CHCl_3_ root extract and the isolated compounds exhibited anticancer activity against L-1210 leukemia cells, with compound II demonstrating a 50% growth inhibition at a low concentration of 4 μg mL^-1^, highlighting the therapeutic potential of *M. dioica* root constituents.

### Anti-microbial properties

Spine gourd exhibits potential as an antimicrobial agent, but the effectiveness depends on two key factors:

Extraction Method:

Researches suggests that a methanolic extract from the fruit displays stronger antimicrobial properties compared to an aqueous extract. Targeted Bacteria and Extract Source: A study explored the antibacterial activity of ethyl acetate extracts from the fruit. They found that a concentration of 200 μg/disc was most effective against *E. coli* compared to other tested bacteria like *S. aureus*, *S. paratyphi*, and *P. mirabilis* (Arekar et al., 2013). Interestingly, the source of the extract also played a role. While both *in vitro* shoot culture (yield: 0.26%) and callus culture (yield: 21.5%) extracts used ethyl acetate, the shoot culture extract showed the highest inhibition zone against *S. paratyphi* and *P. mirabilis*, while the callus culture extract was most effective against *S. aureus* (Arekar et al., 2013).

Methanol is a preferred solvent for extracting antimicrobial compounds from plants due to its versatility in solubilizing a broad spectrum of phytochemicals. Its polarity allows for the extraction of polar metabolites such as flavonoids, alkaloids, and glycosides, while its non-polar characteristics enable the extraction of lipophilic metabolites like terpenoids and steroids. This comprehensive extraction capability often results in methanolic extracts exhibiting superior antimicrobial activity compared to those obtained using other solvents. Additionally, methanol’s inherent antimicrobial properties might contribute to the overall antimicrobial potency of the extract. However, it is essential to consider that the efficacy of a solvent is also influenced by the specific plant species and the nature of the target microorganisms (Yesufu et al., 2022).

It's important to note that Singh et al. found no significant antimycobacterial activity from *Momordica dioica* extracts. Furthermore, research extends beyond the fruit. Some studies suggest that *Momordica dioica* leaf extracts also possess antimicrobial properties, particularly against harmful bacteria such as *E. coli*, *Staphylococcus*, *Pseudomonas*, *Salmonella*, *Streptobacillus*, and *Streptococcus* (Omoregbe et al., 1996).

The concentration and availability of active compounds in plant extracts for antimicrobial studies depend on several factors. The polarity of the solvent plays an important role—polar solvents like water and ethanol are effective at extracting hydrophilic compounds, while non-polar solvents like hexane are more suitable for lipophilic ones (Wakeel et al., 2019). The choice of extraction technique also influences outcomes, as methods such as maceration, percolation, or Soxhlet differ in how efficiently they break down plant tissue and release bioactive metabolites. Other variables, including particle size, drying methods, extraction time, and temperature, affect both the release and preservation of target compounds (Basilio-Cortes et al., 2023). Some bioactive compounds are sensitive to heat or light and may degrade under certain conditions, so adjusting the process is often necessary to retain their activity.

### Analgesic activity

*Momordica dioica* fruit pulp shows promise for pain relief (analgesic), according to multiple studies:

Hexane and Methanol Extracts: Researches (Ilango et al., 2003; Vaidya and Shreedhara, 2003) suggests that both hexane and a specific portion of a methanol extract from the fruit pulp exhibited analgesic effects comparable to a standard pain medication.

Comparative Study: Another study tested the effectiveness of three extracts (petroleum ether, ethyl acetate, and methanol) against acetic acid-induced writhing syndrome in a model system. All three extracts provided significantly more pain relief than the control group. However, petroleum ether and methanol extracts demonstrated a stronger analgesic effect compared to the ethyl acetate extract (Rakh and Chaudhari, 2010).

Here the choice of solvent significantly influences the type of compounds extracted from a plant and consequently, its potential analgesic activity. Although the analgesic activity is often a result of synergistic interactions between multiple metabolites rather than a single isolated molecule. Therefore, the combined effect of different compounds extracted by each solvent contributes to the overall analgesic potential of the plant extract.

Petroleum ether and hexane are non-polar solvents primarily extracting lipophilic compounds like terpenes, steroids, and waxes. Terpenes, especially those with oxygenated functionalities (e.g., terpenoids), have demonstrated analgesic properties due to their interactions with pain receptors or inflammatory pathways (Rose, 2019). Ethyl acetate is a moderately polar solvent capable of extracting a wider range of metabolites including flavonoids, terpenoids, and some alkaloids. Flavonoids have shown analgesic and anti-inflammatory properties, potentially contributing to the extract’s analgesic activity (Al-Khayri et al., 2022). Methanol is a highly polar solvent extracting a broad spectrum of metabolites including alkaloids, flavonoids, glycosides, and phenolic compounds. Alkaloids are known for their diverse pharmacological activities, including analgesia. Additionally, flavonoids and phenolic compounds possess antioxidant and anti-inflammatory properties, which can indirectly contribute to pain relief (Kurek, 2019).

Traditional medicinal uses of spine gourd show substantial alignment with modern pharmacological findings as well as with its nutritional attributes. Ethnomedicinal applications of the crop in the management of diabetes, inflammation, infections, and metabolic disorders are supported by experimental studies reporting antidiabetic, antioxidant, anti-inflammatory, antimicrobial, hepatoprotective, and nephroprotective activities. In addition, spine gourd is nutritionally rich, containing high levels of protein, dietary fiber, essential minerals, vitamins, and health promoting bioactive metabolites, which collectively contribute to its functional food potential. A comparative overview of its nutritional composition relative to commonly consumed vegetables is presented in Table 7, highlighting its relevance for dietary diversification and nutritional security.

**TABLE 7:** Comparative nutritional composition of spine gourd (*Momordica dioica*) and selected commonly consumed vegetables (per 100 g edible portion).

Parameter	Spine gourd	Bitter gourd	Pumpkin (fruit)	Carrot	Tomato	Beetroot
Moisture	84.1%	83.2%–92.4%	91.6 g	88.29 g	91.18 g	91.3 g
Carbohydrates	45%–47.9%	4.2%–9.8%	6.5 g	9.58 g	5.96 g	7.23 g
Protein	18%–19.4%	1.6%–2.9%	1.0 g	0.93 g	17.71 g	1.89 g
Fat	4%–4.7%	0.1%–1%	0.1 g	0.24 g	4.96 g	0.15 g
Dietary Fiber	21.3%–22%	0.8%–1.7%	0.5 g	2.8 g	11.44 g	3.25 g
Calcium (mg)	33–35	20–50	21	33	105	16
Phosphorus (mg)	42–45	70–140	44	35	301	40
Iron (mg)	4–5	2.2–9.4	0.8	0.30	4.55	0.80
Potassium (mg)	8.25	8–170	340	320	403	325
Sodium (mg)	1.51	3–40	1	69	70	78
Magnesium (mg)	Trace	16	12	12	173	23
Zinc (mg)	Trace	0.1	0.32	0.24	2.48	0.365
Manganese (mg)	Trace	0.08–0.32	0.125	0.14	0.60	0.359
Major Bioactives	Unsaturated fatty acids, carotenoids	Charantin, cucurbitane triterpenoids, phenolics	Carotenoids (β and α)	β-carotene, polyphenols	Lycopene, phenolics	Betalains, betaine

Source: Tiwari et al., 2022b; Jha et al., 2017; Gayathry and John, 2022; Batool et al., 2022; Ikram et al., 2024; Ali et al., 2020; Ceclu and Nistor, 2020.

### Critical appraisal of experimental evidence and study limitations

Available studies on *Momordica dioica* and related *Momordica* species collectively indicate considerable nutritional and biological potential, particularly in relation to antioxidant, antidiabetic, anti-inflammatory, and nephroprotective activities. Much of the current evidence is derived from *in vitro* assays and animal models, which are appropriate for early-stage validation and mechanistic insight. These studies provide valuable proof-of-concept support for several traditional and ethnomedicinal claims; however, their findings should be interpreted within the context of experimental conditions, as direct extrapolation to human health outcomes remains limited in the absence of clinical investigations.

A recurring methodological feature across the literature is the use of diverse plant parts, extraction solvents, and preparation protocols, which contributes to variability in phytochemical profiles and reported bioactivities. While this diversity reflects exploratory research efforts, it also complicates cross-study comparison and reproducibility. In several cases, biological effects are reported using crude extracts without full characterization of the active constituents or standardized dosing, making it challenging to establish clear dose–response relationships or identify metabolites responsible for specific effects. Additionally, variations in experimental design, such as treatment duration, sample size, and selection of reference standards, may influence outcome interpretation and highlight the need for greater methodological harmonization.

Based on published studies, the evidence to date is largely foundational in nature. Toxicological assessments, although encouraging in indicating relative safety at certain dose ranges, are not consistently integrated with efficacy studies or aligned with realistic dietary exposure levels. In addition, comparative interpretations across the Momordica genus sometimes rely on evidence from well-studied species such as *M. charantia*, highlighting the importance of species-specific investigation for *M. dioica*. Taken together, the existing literature provides a strong preliminary framework supporting the biological relevance of spine gourd, while also highlighting the need for further validation through standardized experimental designs, compound-level characterization, and clinically relevant models. In this context, the present review serves as a consolidated and critically informed resource that synthesizes dispersed agronomic, nutritional, phytochemical, and bioactivity evidence, clarifies current strengths and limitations, and offers a structured foundation to guide future research, crop improvement strategies, and translational exploration of *M. dioica* as a functional food and underutilized vegetable crop.

### Taxonomic validity and species-level considerations in *Momordica* research

The genus *Momordica* comprises several closely related species that are widely distributed across tropical and subtropical regions and are frequently investigated for nutritional and pharmacological attributes. Accurate species level identification is therefore essential for meaningful interpretation of experimental findings, as interspecific differences in reproductive biology, phytochemical composition, and domestication status may influence biological activity. In the literature, *Momordica dioica* is often discussed alongside more extensively studied species such as *M. charantia*, and while such comparisons can provide valuable biological context, they also necessitate careful taxonomic distinction to avoid overgeneralization across species.

A number of experimental studies included in this review report bioactivities based on authenticated *M. dioica* plant material. Analgesic effects have been demonstrated using hexane and methanolic extracts of *M. dioica* fruit pulp, showing activity comparable to standard analgesic agents under experimental conditions (Ilango et al., 2003; Vaidya and Shreedhara, 2003). Anticancer activity has been reported from chloroform extracts of *M. dioica* roots, where isolated triterpenoid and steroidal compounds, such as α-spinasterol octadecanonate, α-spinasterol-3-O-β-D-glucopyranoside, and related glycosides—were characterized using spectroscopic analyses and shown to inhibit L-1210 leukemia cell growth (Luo et al., 1998). Nephroprotective effects of *M. dioica* methanolic extracts have also been demonstrated in diabetic rat models, with marked improvement in renal histology compared to untreated controls (Gupta et al., 2011).

In addition to species-specific investigations, some studies provide comparative insights between *M. dioica* and other *Momordica* species, particularly *M. charantia*, especially in the context of antidiabetic and metabolic effects. Such comparative evidence is informative for understanding shared and distinct biological traits within the genus and has therefore been consolidated in this review where relevant. However, emphasis has been placed on clearly attributing results to the appropriate species and on prioritizing evidence directly derived from *M. dioica*. This approach allows integration of broader genus-level knowledge while maintaining a focused synthesis of *M. dioica*-specific data and highlights the importance of continued species-resolved investigation and consistent taxonomic reporting in future *Momordica* research.

### Future prospects and research gaps in the pharma and food industry

Spine gourd (*Momordica dioica*) has recently gained significant attention due to its impressive nutritional value and medicinal importance. This perennial dioecious climber is widely used as a vegetable and has a long history of use in traditional medicine, particularly in the management of diabetes and other metabolic abnormalities (Yadav et al., 2024). Phytochemical studies revealed that *M. dioica* contains a wide range of bioactive metabolites, including flavonoids, phenolics, and natural antioxidants, which are believed to contribute to its therapeutic effects (Yadav et al., 2024). Nutritional investigations further indicate that the fruit is high in protein and dietary fiber (accounting for roughly 52% and 15% of its dry weight) along with essential fatty acids and important micronutrients (Salvi, 2015). It also serves as a good source of B-complex vitamins (B1, B2, B6, B9) and minerals such as calcium and magnesium (Salvi, 2015). These findings suggest that *M. dioica* with its broad pharmacological spectrum could serve as a potent dietary supplement, particularly for enhancing nutrition in cereal-based diets in low-resource areas, making it a highly promising resource for both pharmaceutical and nutraceutical applications. (Salvi, 2015; Yadav et al., 2024).

**FIGURE 13:**
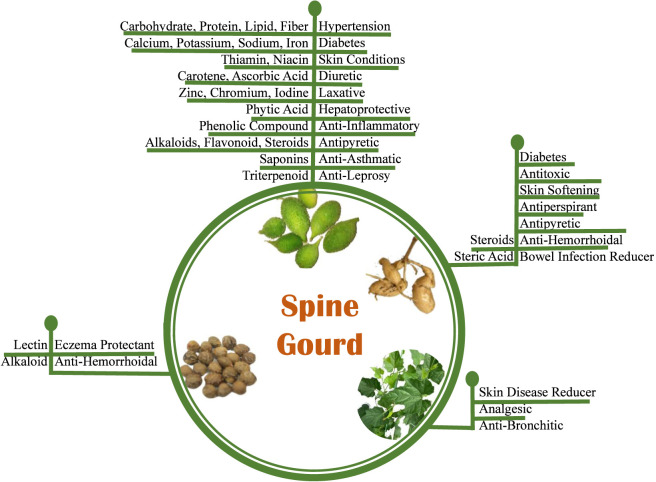
Health benefits of various parts of spine gourd.

Several scientific studies have now confirmed the medicinal claims that have long surrounded the crop. For instance, research has shown that extracts of its fruit have strong antidiabetic effects. In one study, an ethanolic extract from the fruit rind helped improve glucose tolerance in diabetic rats, lowered blood sugar levels after meals, and reduced total cholesterol by around 7% over a 4-week period (Hassan et al., 2022). Its protective effect on kidney function has also been demonstrated. Jain and Singhai (2010) found that treatment with *M. dioica* fruit extract significantly reduced kidney damage caused by cisplatin in rats, most likely due to its high content of natural antioxidants. The plant has also been found to have pain-relieving and anti-inflammatory effects. Different solvent extracts of the fruit pulp (including hexane and ethyl acetate) have been tested in rodents, resulting in reduced pain and inflammation in ways similar to commonly used drugs (Ilango et al., 2003). Moreover, *M. dioica* has shown promising anticancer activity in laboratory settings. In a recent study, an aqueous extract of the fruit was able to inhibit the growth of nearly 50% of human ovarian (PA-I) and cervical (HeLa) cancer cells at a concentration of around 40 μg/mL (Ahirrao, 2019). The plant also has strong antimicrobial properties. Methanolic extracts from its leaves, fruits, stems, and roots showed clear inhibition of several harmful bacteria, including *Staphylococcus aureus*, *Pseudomonas aeruginosa*, *Escherichia coli*, and *Klebsiella pneumoniae* (Ilango et al., 2003). Altogether, these studies provide strong support for its role in developing plant-based treatments for managing diabetes, infections, inflammation, and even cancer. Figure 13 shows the pharmacological uses of the various parts of the spine gourd plant.

In addition to its medicinal use, spine gourd also shows great promise as a nutritious food with added health benefits. The fruit can be eaten fresh or processed into various forms, making it suitable for functional food products. Its high content of fiber and protein may help support digestion and satiety, while its vitamin and mineral composition—particularly vitamins B and K, calcium, and magnesium—can help reduce nutritional deficiencies (Salvi, 2015). The fruit’s fatty acid profile is also favourable for heart health, containing mostly oleic (∼56%) and linoleic (∼22%) acids (Salvi, 2015), which, along with its cholesterol-lowering properties, may contribute to maintaining healthy blood vessels. As plant-based diets and superfoods continue to gain popularity, the combination of nutrients and protective plant metabolites in spine gourd make it a promising ingredient for modern health-conscious consumers. With such a nutritional profile and bioactive richness, the fruit can be utilised for diverse applications in the food industry, including powdered supplements, fortified flours, and functional beverages (Salvi, 2015; Yadav et al., 2024).

To fully make use of spine gourd in both food and pharmaceutical applications, improvements in its cultivation and breeding are essential. Its dioecious nature and low seed germination have long made it difficult to cultivate at scale. However, the use of tissue culture has made it easier to produce female (fruit-bearing) plants in large numbers (Choudhary et al., 2017). Recent studies on genetic variation in both wild and cultivated types have identified a wide range of differences in traits like vine length, fruit weight, yield per plant, seed number, and vitamin C levels, many of which are shown to be heritable and responsive to selection (Yadav et al., 2024). Various breeding techniques are now being explored to improve these traits. For example, gamma radiation has been used to produce hermaphrodite flowers (Tiwari et al., 2024) and improve early plant growth (Tiwari et al., 2019); colchicine treatments have created tetraploid plants with larger leaves and increased pigment content (Nirala et al., 2023); and targeted mutation breeding has resulted in improved plant lines with better fruit yield and desirable traits (Chatterjee et al., 2023). Additionally, root-inducing treatments with IBA have made vegetative propagation more efficient (Tiwari et al., 2022a). These advances show that with continued genetic improvement and agronomic innovation, spine gourd has strong potential to be developed into a reliable crop for health-related industries.

Traditional knowledge can be integrated into scientifically rigorous research by using ethnobotanical information to guide hypothesis based pharmacological, nutritional, and toxicological studies. Documented traditional uses may serve as a foundation for targeted bioactivity screening, followed by *in vivo* validation, standardization of extracts, and safety assessment. Such an evidence based approach can facilitate the development of validated functional foods, nutraceuticals, or phytopharmaceutical products derived from spine gourd.

Spine gourd holds considerable future potential for both the pharmaceutical and food industries owing to its unique combination of nutritional richness, bioactive diversity, and demonstrated biological activities. Accumulating experimental evidence indicates that fruit, leaf, and root extracts of *M. dioica* exhibit antidiabetic, nephroprotective, anti-inflammatory, analgesic, antimicrobial, and anticancer activities under controlled conditions, supporting its long-standing traditional use in managing metabolic and inflammatory disorders (Ilango et al., 2003; Jain and Singhai, 2010; Ahirrao, 2019; Hassan et al., 2022). From a food and nutraceutical perspective, the fruit is particularly promising due to its high protein and dietary fiber content, favourable fatty acid profile dominated by oleic and linoleic acids, and appreciable levels of B-complex vitamins and essential minerals, making it suitable for incorporation into functional foods, fortified flours, dietary supplements, and plant-based health products (Salvi, 2015; Yadav et al., 2024). Advances in breeding, mutation induction, polyploidy development, and micropropagation have further improved prospects for large-scale cultivation by addressing constraints related to dioecy, low seed germination, and yield variability (Tiwari et al., 2019; Tiwari et al., 2022b; Tiwari et al., 2024; Chatterjee et al., 2023; Nirala et al., 2023). Integrating these agronomic and genetic improvements with ethnobotanical knowledge and rigorous pharmacological validation offers a clear pathway for translating spine gourd from an underutilized traditional crop into a scientifically substantiated source of functional foods, nutraceuticals, and phytopharmaceutical products.

While *Momordica dioica* shows substantial promise for applications in the food, nutraceutical, and pharmaceutical sectors, realizing its full potential will require addressing several research and development gaps. Most available evidence is derived from *in vitro* or animal-based studies using variable plant material and extraction protocols, highlighting the need for standardized phytochemical characterization, dose response validation, and safety assessment under realistic dietary exposure levels. The absence of human clinical studies, particularly for metabolic, renal, and inflammatory indications, remains a key limitation for translational advancement. Future efforts should integrate agronomic improvement, genomics assisted breeding, and metabolomic profiling with pharmacological and nutritional research to link genetic diversity with functional traits and bioactive composition. Strengthening conservation strategies, developing structured planting material systems, and aligning traditional knowledge with rigorous experimental validation will further support the sustainable commercialization of spine gourd as a clinically validated functional food and phytopharmaceutical resource.

## Conclusion

This review provides a comprehensive and critically informed synthesis of existing knowledge on spine gourd (*Momordica dioica* Roxb.), integrating evidence on its traditional uses, nutritional composition, phytochemical profile, agronomic traits, and experimentally reported bioactivities. Available studies consistently indicate that *M. dioica* is nutritionally rich and contains diverse classes of bioactive metabolites that support several ethnomedicinal applications, particularly those related to metabolic regulation, antioxidant capacity, and inflammation. At the same time, a clear distinction emerges between exploratory observations and well substantiated findings, as much of the reported pharmacological activity has been demonstrated under controlled experimental settings rather than through standardized validation.

Beyond its biological potential, spine gourd represents a promising yet underexploited crop with adaptability to diverse agroclimatic conditions and relevance for functional food and nutraceutical development. However, its wider domestication and commercialization remain constrained by biological and agronomic limitations, including dioecy, low seed viability, and restricted multiplication rates. Existing research efforts are often fragmented across disciplines, with limited emphasis on coordinated breeding strategies, genetic improvement, and systematic deployment of modern propagation technologies. Addressing these constraints through targeted research on sex determination, yield stabilization, vegetative and *in vitro* propagation, and molecular characterization is essential for advancing the crop beyond niche cultivation.

A key contribution of this review is its integrative and evaluative perspective, which consolidates dispersed literature while explicitly distinguishing between preliminary experimental evidence and outcomes with greater translational relevance, and situates *M. dioica* within both a species-specific and comparative *Momordica* framework. By linking ethnobotanical knowledge with experimental findings, agronomic constraints, and emerging breeding and biotechnological advances, this synthesis identifies clear research priorities and translational pathways. Future interdisciplinary efforts spanning plant breeding, genomics, food science, pharmacology, and toxicology will be crucial for translating the nutritional and therapeutic promise of *M. dioica* into evidence-based food and health applications, thereby supporting its transition from an underutilized traditional vegetable to a sustainable and economically viable component of resilient food systems.
